# Summer-Wet Hydrologic Cycle during the Middle Miocene of the United States: New Evidence from Fossil Fungi

**DOI:** 10.34133/research.0481

**Published:** 2024-09-24

**Authors:** Jennifer M. K. O’Keefe, Matthew J. Pound, Ingrid C. Romero, Noelia B. Nuñez Otaño, Martha E. Gibson, Jessica McCoy, Margaret E. Alden, C. Jolene Fairchild, Julia Fitzpatrick, Emily Hodgson, Taylor Horsfall, Savannah Jones, June E. Lennex-Stone, Christopher A. Marsh, Alyssa A. Patel, Tyler M. Spears, Laikin Tarlton, Liberty F. Smallwood, O. L. VanderEspt, Jeremyah R. Cabrera, Cortland F. Eble, William C. Rember, James E. Starnes, Mac H. Alford, Alyson Brink, Sophie Warny

**Affiliations:** ^1^ Department of Engineering Sciences, Morehead State University, Morehead, KY, USA.; ^2^ Department of Geography and Environmental Sciences, Northumbria University, Newcastle upon Tyne, UK.; ^3^ Department of Paleobiology, National Museum of Natural History, Smithsonian Institution, Washington, DC, USA.; ^4^ Laboratorio de Geología de Llanuras (CICYTTP– FCyT), CICYTTP (CONICET-Prov. ER—UADER), Diamante, Entre Ríos, Argentina.; ^5^ CONICET (National Scientific and Technical Research Council), Buenos Aires, Argentina.; ^6^ PetroStrat, Conwy, Wales, UK.; ^7^ Department of Biology and Chemistry, Morehead State University, Morehead, KY, USA.; ^8^ Craft Academy for Excellence in Science and Mathematics, Morehead State University, Morehead, KY, USA.; ^9^ Department of Agricultural Science, Morehead State University, Morehead, KY, USA.; ^10^ Kentucky Geological Survey, Lexington, KY, USA.; ^11^ Independent Researcher, Fernwood, ID, USA.; ^12^ Mississippi Department of Environmental Quality, Office of Geology, Jackson, MS, USA.; ^13^ School of Biological, Environmental, and Earth Sciences, University of Southern Mississippi, Hattiesburg, MS, USA.; ^14^ Center for Excellence in Palynology, Department of Geology & Geophysics, and Museum of Natural Science, Louisiana State University, Baton Rouge, LA, USA.

## Abstract

Hydrologic reconstructions from North America are largely unknown for the Middle Miocene. Examination of fungal palynomorph assemblages coupled with traditional plant-based palynology permits delineation of local, as opposed to regional, climate signals and provides a baseline for study of ancient fungas. Here, the Fungi in a Warmer World project presents paleoecology and paleoclimatology of 351 fungal morphotypes from 3 sites in the United States: the Clarkia Konservat-Lagerstätte site (Idaho), the Alum Bluff site (Florida), and the Bouie River site (Mississippi). Of these, 83 fungi are identified as extant taxa and 41 are newly reported from the Miocene. Combining new plant-based paleoclimatic reconstructions with funga-based paleoclimate reconstructions, we demonstrate cooling and hydrologic changes from the Miocene climate optimum to the Serravallian. In the southeastern United States, this is comparable to that reconstructed with pollen and paleobotany alone. In the northwestern United States, cooling is greater than indicated by other reconstructions and hydrology shifts seasonally, from no dry season to a dry summer season. Our results demonstrate the utility of fossil fungi as paleoecologic and paleoclimatic proxies and that warmer than modern geological time intervals do not match the “wet gets wetter, dry gets drier” paradigm. Instead, both plants and fungi show an invigorated hydrological cycle across mid-latitude North America.

## Introduction

The Miocene climate optimum [MCO; 16.9 to 14.7 million years ago (MA)], a period of warmer-than-present climates globally, provides a proxy for likely future warming [1]. While global temperatures of the MCO are well established [1,2], the hydrological cycle is still poorly understood. In North America, recent fossil plant-based reconstructions suggested that rainfall patterns were more seasonal than modern [3]. Here, we use a recently developed fossil fungi paleoclimate proxy to reconstruct Köppen-Geiger climate classifications [4] and use it to qualitatively test this “more seasonal than modern” hypothesis.

Both extant fungal biogeography and previous works examining fossil fungal assemblages have shown that they are sensitive to ambient climate [4–6].

Using fungal remains preserved during and following the MCO across a latitudinal gradient to reconstruct climate provides a fossil plant-independent perspective on past hydrological conditions.

**Fig. 1.**
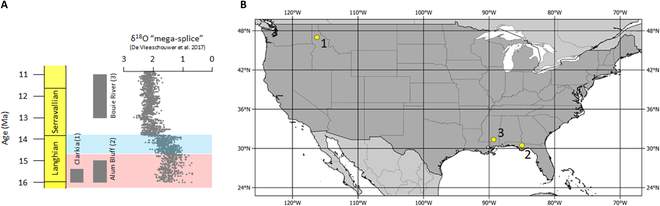
Fungal study site ages and location. (A) Age of the 3 study sites plotted in relation to the δ18O “mega-splice” [2]. (B) Location of the 3 study sites within the United States. Blue shaded zone indicates Middle Miocene Climate Transition (MMCT), while pink shaded zone indicates Miocene Climate Optimum (MCO).

Alum Bluff (Florida) and the Bouie River (Mississippi) site, located in the southeastern part of the United States, provide a record from 16 to 11.6 MA [3], encompassing part of the MCO (Alum Bluff), and the interval of time after the global cooling of the Middle Miocene climatic transition (MMCT) at Bouie River (Fig. 1; Supplementary Materials, SI1). The Clarkia Konservat-Lagerstätte (northern Idaho), located in the northwestern part of the country, provides a well-constrained record from the MCO (Fig. 1; Supplementary Materials, SI1). Trapper Creek (southern Idaho) provides a paleobotanical record of Serravallian climate for the northwest but was not part of the fungal study (Fig. 1). Existing paleobotanical and palynological records for these sites [7–12] suggest that a mosaic of plant communities inhabited the temperate to tropical Alum Bluff, subtropical to temperate Bouie River, warm-temperate Clarkia sites, and cool-conifer forests of Trapper Creek [13]. These 4 sites create a latitudinal and temporal perspective on North American paleoclimates during this warmer-than-present world.

Fossil fungal remains have been previously documented from all sites and have been the subject of minor studies at both Alum Bluff and Clarkia. Jarzen et al. [7] identified fungi present at Alum Bluff using fossil names, which have little correlation with modern taxonomic names and thus could not, as such, contribute ecological or climatological information about the fungi present. Most fungal taxa described from Clarkia to date utilize modern names; however, the focus has been primarily on epiphyllous fungi, which are not frequently encountered in palynological preparations from this site [8,14–16]. This is the first study to systematically extract, identify, and interpret fossil fungi preserved in sediments from Clarkia, Alum Bluff, and the Bouie River site (Fig. 1) in the full context of their modern counterparts. Where possible, fossil taxa were identified using modern names, which facilitates their use for paleoclimate reconstructions. These fungal-based reconstructions were then compared to new quantitative paleobotanical reconstructions to better elucidate both local and regional climate signals at each locality.

## Results

**Fig. 2.**
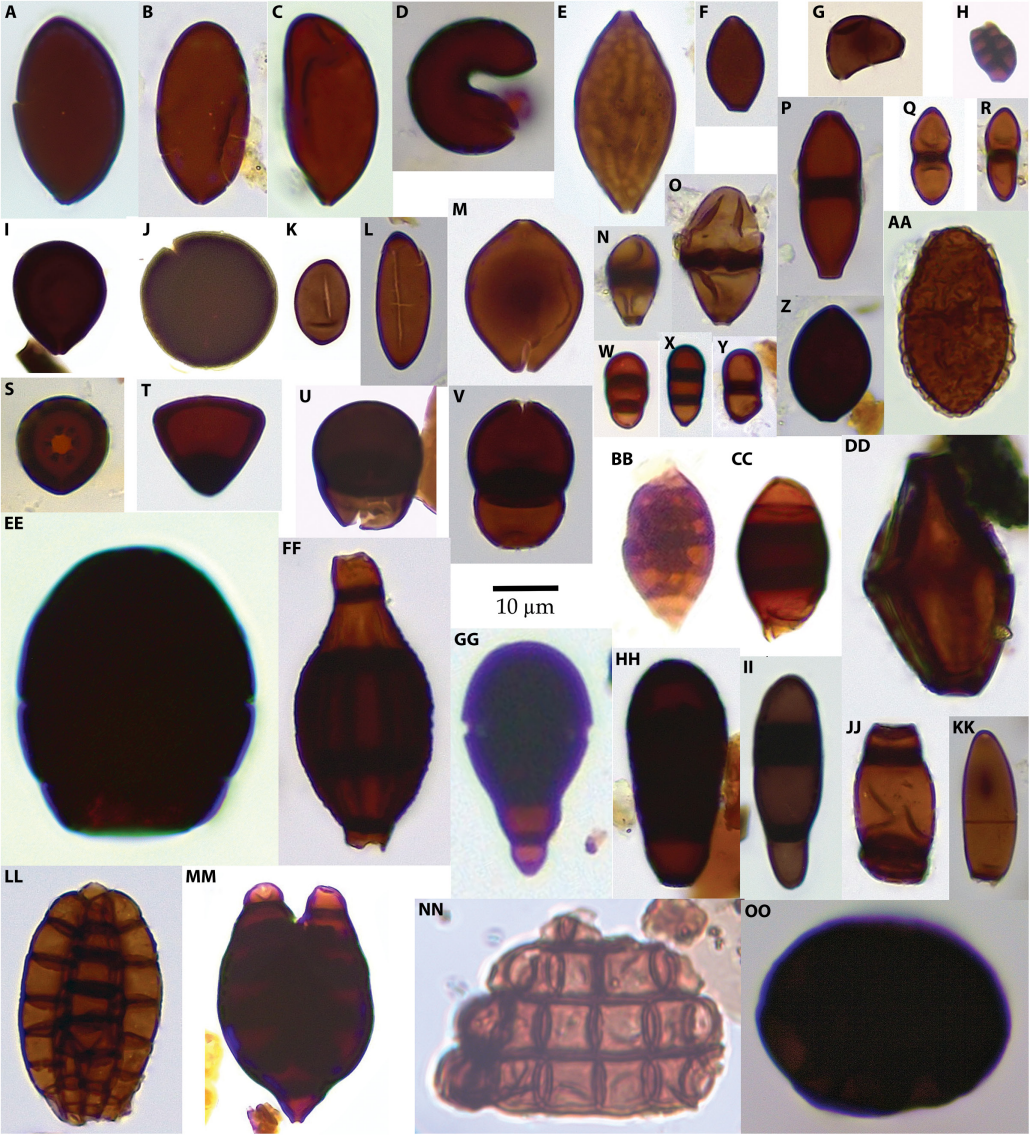
Fungal palynomorphs recovered from the Clarkia Konservat-lLagerstätte. (A) cf. *Bombardioidea*. (B) cf. Sordariales. (C) cf. *Brachydesmiella*. (D) cf. *Melanographium*. (E) *Sphaerodes*. (F) cf*. Cercophora*. (G) cf. *Polytretophora*. (H) *Hughesinia*. (I) cf. *Acrogenospora*. (J) cf. Apiosporaceae. (K) Xylariaceae. (L) cf. *Rosellinia*. (M) *Chaetomium*. (N) *Acarocybiopsis*. (O) *Endophragmia*. (P) *Bispora*. (Q) cf. *Didymosphaeria*. (R) *Neoroussoella*. (S) cf. *Lemkea*. (T) cf. *Catenularia*. (U) *Endophragmiella*. (V) *Desertella*. (W) *Bactrodesmium*. (X) *Spadicoides* sp. 1. (Y) *Spadicoide*s sp. 2. (Z) *Podospora*. (AA) *Minutisphaera*. (BB) *Pendulispora*. (CC) *Phragmocephala*. (DD) *Diporotheca* aff. *rhizophila*. (EE) *Melanocephala*. (FF) *Seychellomyces*. (GG) *Trichocladium*. (HH) *Brachysporiella*. (II) *Bactrodesmium* aff. *abruptum*. (JJ) *Endophragmiopsis*. (KK) *Naviculispora*. (LL) *Dictyocheirospora*. (MM) *Helensiella*. (NN) cf. *Helicoon*. (OO) cf. *Hermatomyces*. Scale bar, 10 μm.

**Fig. 3.**
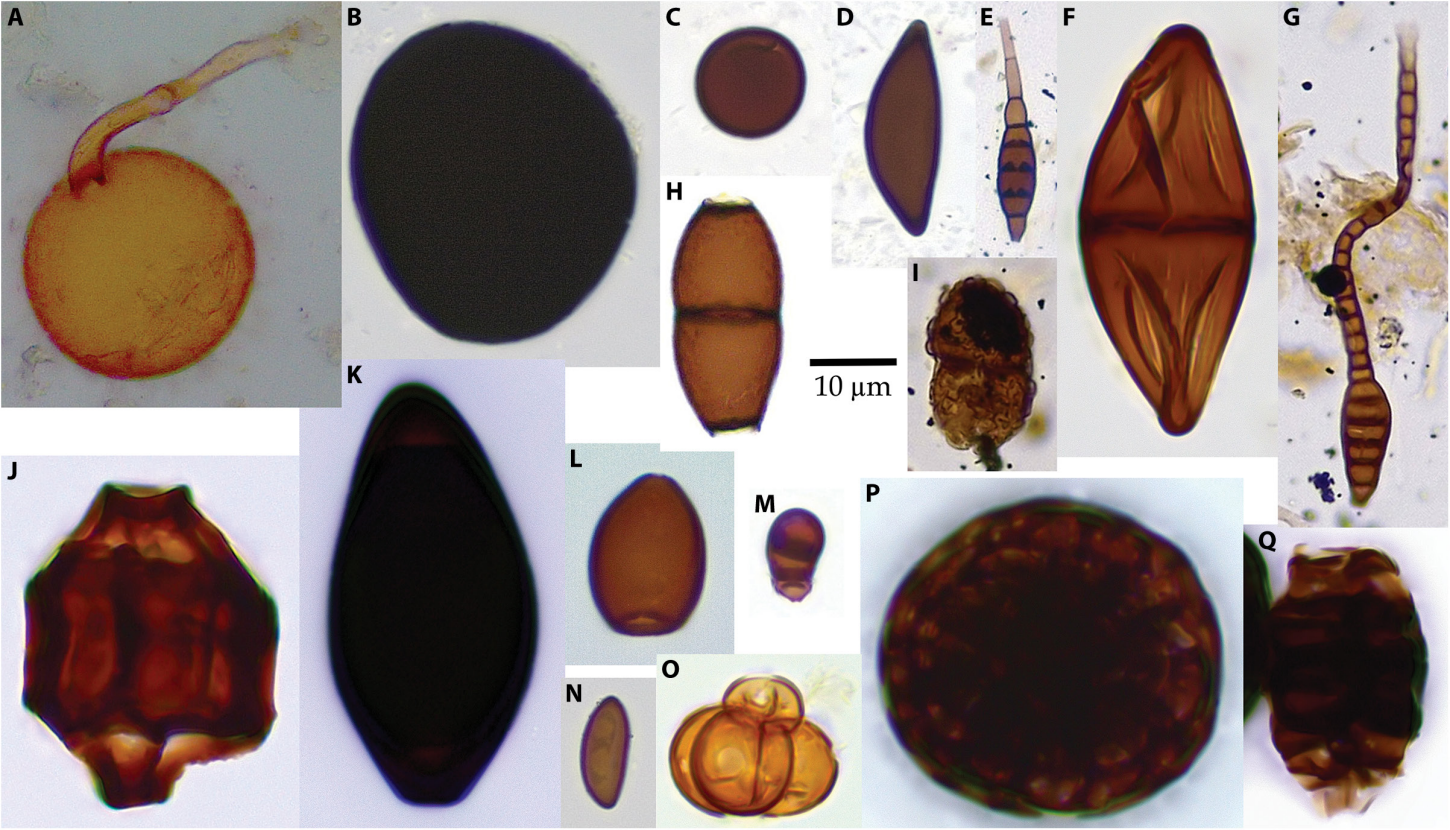
Fungal palynomorphs recovered from Alum Bluff. (A) Glomeromycota. (B) *Acrogenospora*. (C) Apiosporaceae. (D) *Rosellinia*. (E) *Elegantimyces*. (F) *Atrotorquata* aff. *lineata*. (G) *Sporidesmium*. (H) *Savoryella* aff. *lignicola*. (I) *Minutisphaera*. (J) *Sphaerodes*. (K) cf. *Kionocephala*. (L) *Cercophora*. (M) *Spadicoides*. (N) Xylariales. (O) *Thecaphora*. (P) cf. *Hermatomyces*. (Q) *Canalisporium*. Scale bar, 10 μm.

**Fig. 4.**
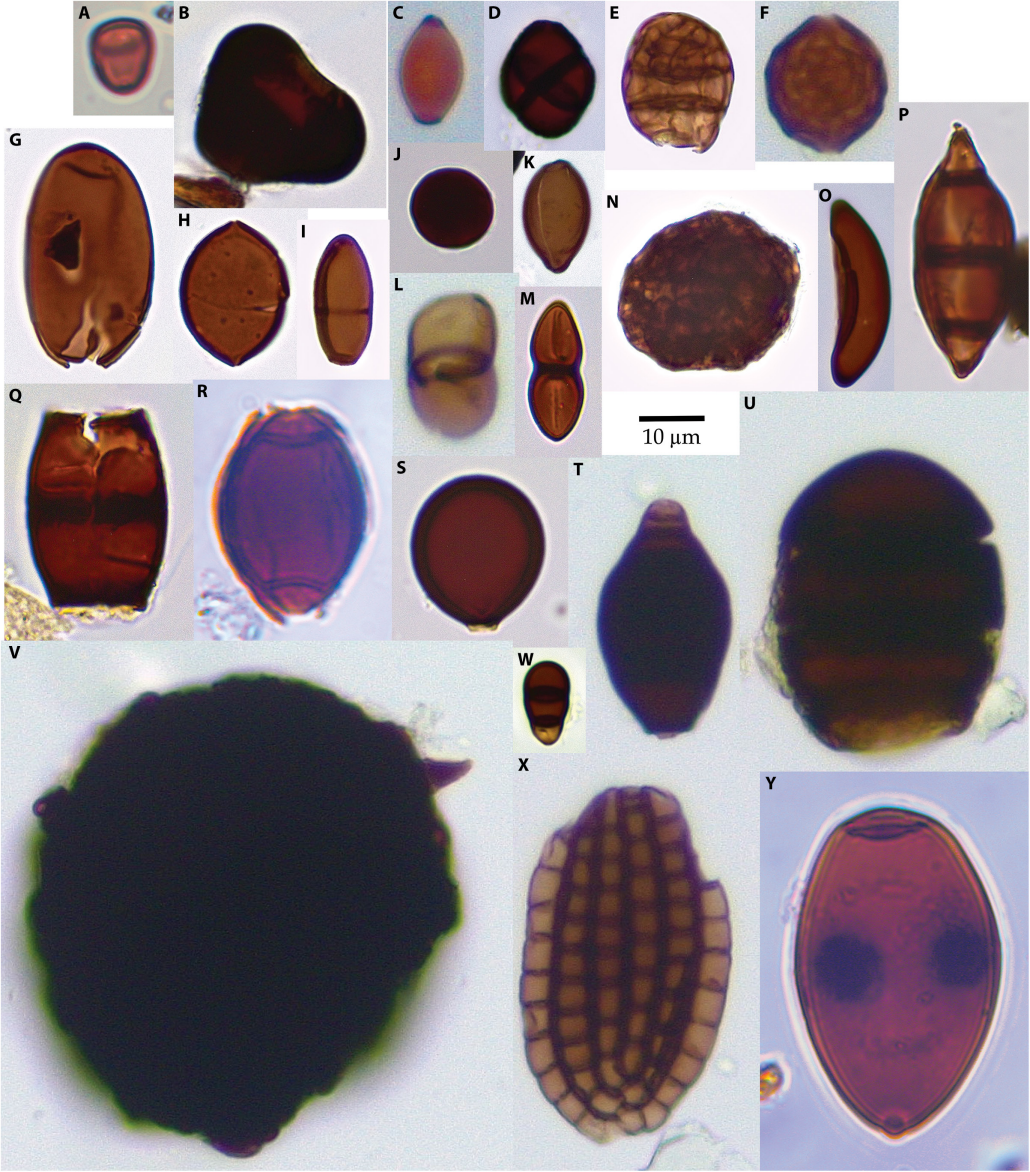
Fungal palynomorphs recovered from the Bouie River site. (A) cf. *Catenularia*. (B) *Zopfiella* aff. *neogenica*. (C) cf. *Cercophora*. (D) *Spegazzinia* β-type. (E) cf. *Bahugada*. (F) *Sphaerodes*. (G) Sordariales. (H) *Chaetomium*. (I) cf. *Naviculispora*. (J) Apiosporaceae. (K) Xylariales. (L) cf. *Asterina*. (M) cf. *Delitschia*. (N) cf. *Berkleasmium*. (O) cf. *Rosellinia*. (P) cf. *Caryospora*. (Q) cf. *Endophragmiella*. (R) cf. *Diporotheca*. (S) cf. *Acrogenospora*. (T) cf. *Heteroconium*. (U) cf. *Melanocephala*. (V) *Termitariopsis*. (W) cf. *Bactrodesmium*. (X) cf. *Dictyosporium*. (Y) *Potamomyces.* Scale bar, 10 μm.

A total of 351 fungal morphotypes were obtained from the 3 sites (Supplementary Materials, SI2). Eighty-three of the morphotypes have been identified as modern taxa. Of these, 41 genera have not previously been noted in the Miocene: cf. *Acarocybiopsis*, *Bispora*, *Bombardioidea*, *Brachydesmiella*, cf. *Desertella*, *Dictyocheirospora*, *Didymosphaeria*, cf. *Helensiella*, *Hughesinia*, *Massaria, Melanographium*, cf. *Neoroussoella*, *Pendulispora*, cf. *Polytretophora*, and *Seychellomyces* are unique to the Clarkia Lagerstätte; cf. *Alysidium*, *Atrotorquata* aff. *lineata*, cf. *Cladosporium*, *Gliomastix* aff. *fusigera*, cf. *Kionocephala*, *Puccinia-*type, *Sporidesmium*, *Thecaphora*, and cf. *Trimmatostroma* are unique to Alum Bluff; cf. *Bahugada*, *Caryospora*, *Heteroconium*, cf. *Melanospora*, *Septonema*, *Spegazzinia* β-type, and cf. *Termitariopsis* are unique to the Bouie River site; *Brachysporium*, cf. *Endophragmiopsis*, *Melanocephala*, cf. *Naviculispora*, and *Trichocladium* occur in both the Bouie River site and the Clarkia Konservat-Lagerstätte; *Hermatomyces*, *Minutisphaera*, and *Sphaerodes* occur in both Alum Bluff and the Clarkia Konservat-Lagerstätte; and *Chaetomium* and *Spadicoides* occur in all 3 sites. Samples from Clarkia (Fig. 2) generally contained the most diverse fungal assemblages, with up to 37 morphotypes present in any sample. The lowest fungal diversity occurred near the bases of ash deposits, especially in ash RA-3. Samples from Alum Bluff (Fig. 3) generally contained low-diversity fungal assemblages, with an average of 4 taxa in the lower portion of the measured section and 2 taxa in the upper portion of the measured section. The Jarzen et al. [7] samples, thought to be from just above the measured section, had somewhat higher diversity than those from the lower portion of the measured section. Samples from the Bouie River site (Fig. 4) exhibited similar low diversity.

### Paleoecology

Most taxa recovered from the Clarkia Konservat-Lagerstätte are wood saprophytes (Supplementary Materials, SI2 and SI3). All trophic modes are represented except keratinophytic, animal parasitic, and nematophagous. Fungi recovered are largely indicative of freshwater terrestrial environments that are variably submerged or subaerial. Examination of fungal guild structure is limited by 2 factors: (a) nonrecovery of very fragile and hyaline fungal spores and (b) the large number of taxa that could not be confidently assigned to a modern analog within each sample. To largely circumvent these challenges, only those samples that contained greater than 10 identified fungi were included in the analysis for Clarkia and subsequent settings. In this limited subset, 3 variations in guild structure were noted: (a) saprotroph dominated with near-equal numbers of dark septate endophytes (DSEs) and unknowns, and a greater number of pathogenic fungi (Supplementary Materials, SI4); (b) saprotroph-unknown co-dominated; and (c) saprotroph-unknown co-dominated with DSEs and pathogenic fungi present. Wood saprophytes are also common in sediments from Alum Bluff (Supplementary Materials, SI2 and SI3), and keratinophytic, hyperparasitic, and nematophagous modes are absent. Fungi recovered are generally indicative of freshwater conditions, although marine and/or brackish water elements are present at various points in the column (e.g., samples 43592, 1499, 1495, 1486, and 1482). Fungal guild structure reconstructions are not possible for Alum Bluff due to low taxonomic diversity in individual samples. The Bouie River site is especially challenging to characterize as very few samples contain many identifiable fungi (Supplementary Materials, SI2 and SI3). That said, the only trophic mode not present in the fungas from the Bouie River site is nematophagous. Marine/Brackish water elements are more likely in the lower third of the section than elsewhere. Only one sample (1516) could be analyzed for guild structure. The funga present in this sample is remarkably similar to the third type noted above for Clarkia (Supplementary Materials, SI4).

### Paleoclimatology

#### Köppen-Geiger classes

Köppen-Geiger class reconstructions using fungi are noisy where primarily cosmopolitan taxa are present [4]. This is the case for large parts of the reconstructions from the Clarkia Konservat-Lagerstätte, the Alum Bluff site, and the Bouie River site (Supplementary Materials, SI4).

**Fig. 5.**
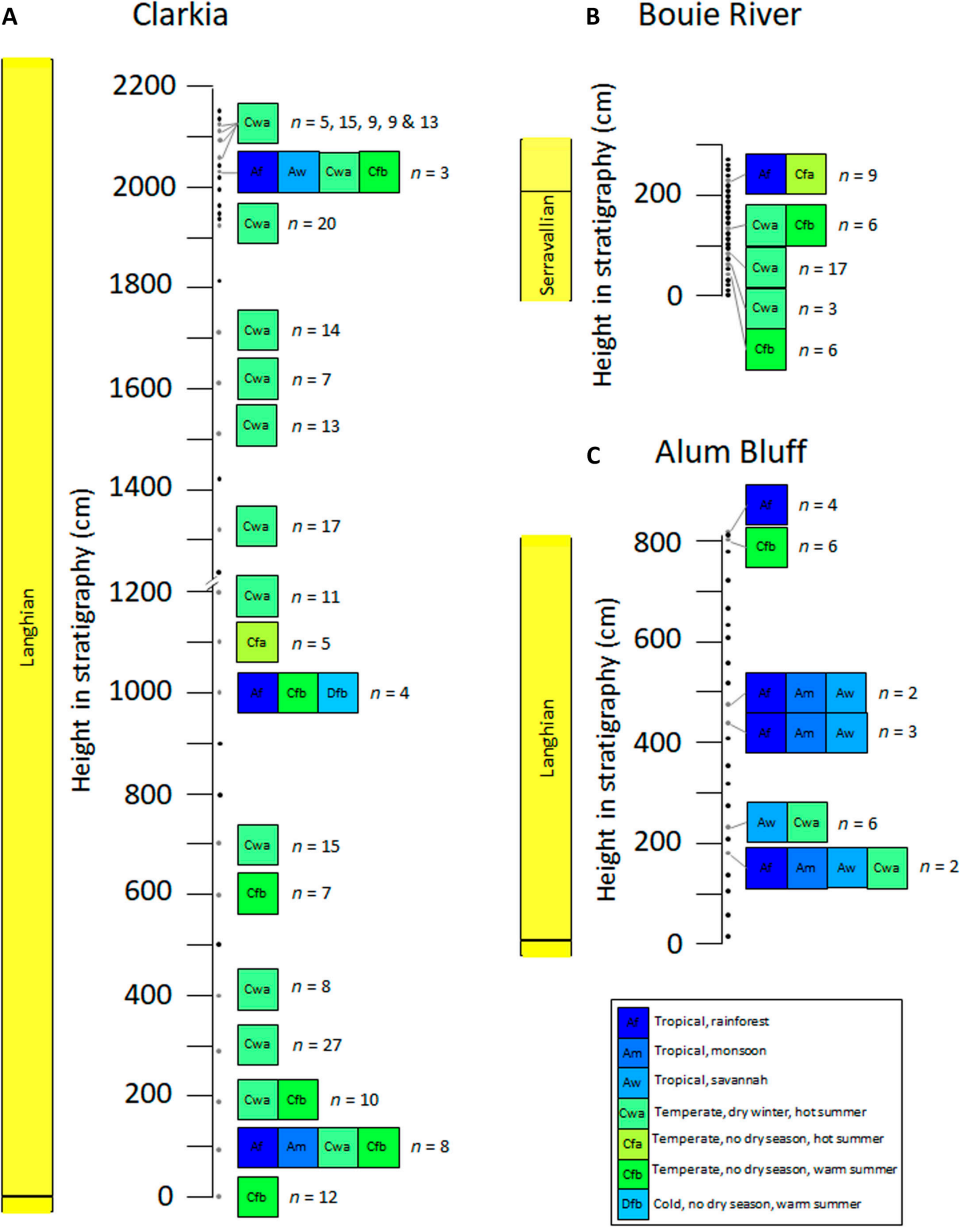
Funga-based Köppen-Geiger climate class reconstructions for (A) Clarkia, (B) Bouie River, and (C) Alum Bluff.

In the Clarkia Konservat-Lagerstätte (Fig. 5), the fungal-indicated Köppen-Geiger class at the time of lake initiation at P-33 (sample 1501) is temperate with no dry season and warm summers (Cfb). While noisy, the next 3 m records a transition to temperate with dry winters and hot summers (Cwa). A brief return to Cfb conditions is seen 2 m above this before reverting to Cwa. At 11 m, a temperate with no dry season and hot summers (Cfa) climate is indicated, largely due to the presence of *Brachyporiella* aff. *setosa*, before a return to Cwa conditions. The entirety of site P-37, where single Köppen-Geiger classes could be reconstructed, was likely deposited under Cwa conditions.

In stratigraphically ordered samples from the Alum Bluff site (Fig. 5), no single Köppen-Geiger class can be clearly reconstructed from the fungi. The most constrained reconstruct tropical rainforest, monsoonal, or savannah with dry winters (Af, Am, Aw) or Aw to temperate with dry winter and hot summer (Cwa) conditions. The lowermost 5 productive samples may record a transition from primarily tropical to primarily warm temperate conditions. While the remaining samples in the stratigraphic section are noisy, they consistently reconstruct Am and Aw climates, suggesting a return to tropical conditions. In the Jarzen et al. [7] samples, treated as being from similar horizons above the measured section, reconstructions range from Af or Am to mixed Aw and Cfb to Cfa or Cwa conditions.

At the Bouie River site (Fig. 5), the fungal-indicated Köppen-Geiger classes range from Cfb (1506, 1508) to Cwa (1512, 1514, 1516), mixed Cwa and Cfb at 1.3 m (1526), and mixed Af and Cfa at 2.2 m (1542), suggesting that subtropical wet conditions predominated.

#### Paleobotany-based reconstructions using crestr

**Fig. 6.**
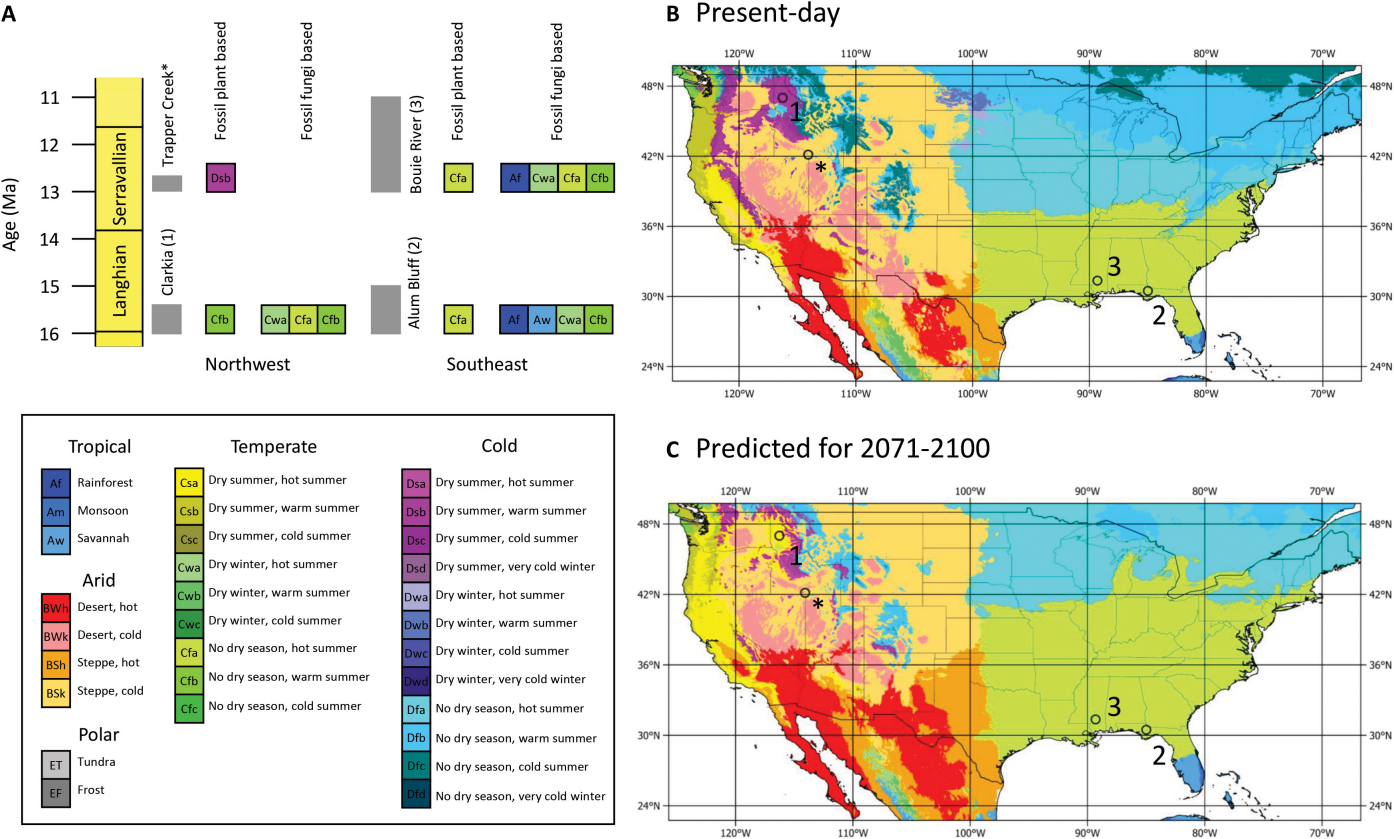
Comparison of (A) fossil plant and fossil funga-based Köppen-Geiger climate class reconstructions with (B) present-day distributions of Köppen-Geiger climate classes and (C) Köppen-Geiger climate class distributions predicted for 2071–2100 in the contiguous United States ([36]; Supplementary Materials, SI5).

Our new plant-based paleoclimate-defined Köppen-Geiger classifications show that Bouie River and Alum Bluff supported a flora in a Cfa (temperate, no dry season, hot summer) class, whereas Clarkia and Trapper Creek were reconstructed as Cfb (temperate, no dry season, hot summer) and Dsb (cold, dry summer, warm summer) classes, respectively (Fig. 6; Supplementary Materials, SI5). These reconstructions show a clear climatic cooling and increase in seasonality between the 42°N to 47°N MCO Clarkia and post-MMCT Trapper Creek. This cooling was on the order of 3.65 °C for mean annual temperature (MAT) and summer temperature and 2.25 °C in winter (Supplementary Materials, SI5). Although the Köppen-Geiger class of Alum Bluff and Bouie River are the same, a 1.15 °C drop in MAT and a 2.35 °C decrease in winter temperature are reconstructed (Supplementary Materials, SI5). All new reconstructions are comparable to previously published estimates (Supplementary Materials, SI6).

## Discussion

The MCO has been considered an analog for future climate change [1]. Here, we compare our new results to predictions for end-21st century Köppen-Geiger climate class distribution. In North America, the distribution of Köppen-Geiger classes largely follows the “dry gets drier, wet gets wetter” paradigm that assumes that higher temperatures will exaggerate contemporary hydrological conditions, which has been shown to be an invalid assumption for over 70% of land areas [17]. For the warmer-than-present MCO, our results show wetter conditions, with fossil fungi showing a summer-wet hydrological regime (Fig. 6). A summer-wet regime characterizes many present-day monsoon regions, including the North American Monsoon [18]. Presently, the North American Monsoon only delivers infrequent heavy rainfall events to the southern United States during summer and is projected to change by ±5% with contemporary CO_2_ increase [18,19]. This is supported by middle Miocene climate modeling that showed a weaker North American Monsoon [20]. Atmospheric rivers are projected to increase winter rainfall across western North America with future climate change [21]. This may be a causal mechanism for the winter-wet climate reconstructed for Trapper Creek during the Serravallian, but not the aseasonal (plant-based) or summer-wet (fungal-based) climate reconstructed for MCO Clarkia (Fig. 6). Increased mean annual precipitation is a consistent feature of the MioMIP1 multi-model ensemble in western North America and increases in geographic area with increasing atmospheric CO_2_; however, it is currently unknown how this responds seasonally [20]. Additionally, uplift of the Cascade Range and portions of the Rocky Mountains in the middle to late Miocene has fundamentally changed the hydrologic regime in what is now north-central Idaho [22], and mid-Miocene climate reconstructions are unlikely to fully reflect the impact of future warming scenarios for this region. Our reconstructed post-MCO cooling in the northwest is greater than that reported in Greenwood et al. [23]. Taggart and Cross [13] proposed Trapper Creek as a cold conifer forest, which is supported by our Köppen-Geiger reconstruction. In southeast North America, the plant-based paleoclimate reconstruction shows the presence of the same Köppen-Geiger classification as today (Fig. 6), and that which is predicted to be present to the end of the 21st century and is consistent with other palaeobotanical reconstructions for these sites [3]. The fungal-based reconstructions are more uncertain due to poorer preservation, but also point to a warmer and potentially wetter climate during the middle Miocene. This is supported by climate model studies, although this is also a region with high model disagreement for the hydrologic response [20].

Reconstructions based on fungi and plants, while in this case complementary, are fundamentally completed at different scales and focused on different aspects of the system. Macro-paleobotany-based reconstructions are, by nature of the defined assemblages, time-averaged, as they represent accumulation across much broader stratigraphic intervals than microfossil-based reconstructions [24]. Plant palynology data, with few exceptions, reconstructs a regional signal [24] due to admixture of long-transport and local pollen sources. Fungal palynology, due to the relatively small dispersal distance of most fungi (millimeters to meters), provides a highly local signal in endorheic lakes and peat-producing settings [25,26]. While transport distances can be greater in alluvial-fluvial settings, the signal remains more localized than that of anemophilous pollen. Additionally, the taphonomy of fungi differs from that of both leaves and plant pollen and spores due to the chemistry of the setting and the chemistry of the fungal palynomorphs. Melanized fungal remains are more resistant to degradation by both bacteria and other fungi [27,28], and are thus more likely to be preserved in the fossil record. Darkening of weakly melanized fungal spores is known to occur through a variety of mechanisms [27] and to vary with thermal maturity [29]. All samples examined in this study are thermally immature; therefore, that factor can be discounted. Beyond mere preservation, the nature of fungal palynomorphs as a biological entity is very different from plant palynomorphs. Plant palynomorphs that fall on the ground are reproductively sterile—they will not progress further along their life cycles and are largely preserved as whole organs. Fungal palynomorphs, however, represent fungal necromass—for whatever reason, conditions were inhospitable for continuance of the fungal life cycle [25]. We, therefore, expect to see more limited and highly localized assemblages in the fossil record. Thus, we are comparing a taphonomically distinct local signal to a regional and/or time-averaged signal when comparing fungi-based to plant-based reconstructions.

### Conclusions

Three hundred fifty-one fungal morphotypes were recorded from Miocene sections in North America, 83 of which were identified as extant forms, and 41 of which were not previously known from the Miocene. While recovery varied from very good (Clarkia) to sparse (Alum Bluff), the recovered fungas permitted interpretation of both paleoecology and paleoclimate.

Fungas recovered from Miocene-aged sediments from Clarkia, Idaho, Alum Bluff, Florida, and the Bouie River site, Mississippi, complement plant-based paleoclimate reconstructions. At Clarkia, the MCO fungal-reconstructed Köppen-Geiger classes fluctuate from a dominant Cwa to Cfa and Cfb, showing a warm-temperate to temperate climate with dry winters or no dry season that is consistent with the fossil pollen-based reconstructions. Following the MCO, fossil plant reconstructions show a cooling to a cold climate with a dry and warm summer (Dsb), indicating a hydrological shift in response to either global cooling, regional uplift, or a combination of the 2. In southeastern North America, the fungal-based reconstructions are hampered by poor preservation but still produce results consistent with paleobotany. At Alum Bluff, the reconstruction ranges from seasonal tropical/subtropical (Aw/Cwa) to aseasonal tropical (Af) leading up to and during the MCO, and Af/Cfa/Cwa/Cfb for the Bouie River site following the MCO. The numerical paleobotanic reconstructions show a 6 °C MAT cooling in Idaho (42°N to 46°N) and 1 °C cooling in the southeast (30°N to 31°N) from the MMCT. Overall, these fungal-based results are consistent with paleobotanical reconstructions and show a wetter middle Miocene in North America. This provides further geological evidence that contradicts the “wet gets wetter, dry gets drier” paradigm of elevated global temperatures.

## Materials and Methods

### Sample collection and palynological processing

Samples were collected from freshly excavated vertical trenches. At Clarkia and Alum Bluff, individual samples were obtained in 2.5-cm-diameter 15-cm-long sterile polyvinyl chloride tubes pounded into the exposure; in cases where this was not feasible, a stainless-steel grain scoop of similar depth was used to obtain samples. At the Bouie River site, rising water levels dictated collection of pillar samples from a step trench. The pillar samples were subsampled for study upon return to the laboratory. All samples were dried in a 50 °C oven and crushed to −1 mm prior to processing. Processing followed hydrofluoric acid (HF)-free protocols outlined by O’Keefe and Eble [30] and Pound et al. [31], except for select samples from Alum Bluff, which were treated with multiple rounds of microwave-assisted HF digestion following HCl treatment to remove carbonates.

### Fungal palynology

Microscopy and image collection followed the methods of Romero et al. [6]. Images were sorted into OneNote files based on palynomorph morphology, and taxonomic identifications were completed using the morphometric methods of Pound et al. [4] as well as comparisons with materials from The Fungarium at Royal Botanic Gardens Kew (Supplementary Materials, SI2). Unidentified specimens were assigned the prefix OPaL (the paleoecology laboratory at Morehead State University) and a number, following the recommendations of O’Keefe et al. [32] and Miola [33] to avoid increasing taxonomic instability caused by giving a likely extant organism a fossil name. Occurrence data were tabulated; counts were not performed following the recommendations of Perrotti et al. [34] and Nuñez Otaño et al. [25]. Fungal traits and geographic distributions were determined following the methods of Pound et al. [4] in April 2023 (Supplementary Materials, SI3). Fungal Guild assignments were made via r-script query of FUNGuild [35] in March of 2024; for those taxa not listed in FUNGuild, the primary literature was consulted to derive guild assignments (Supplementary Materials, SI4). Qualitative paleoclimate reconstructions of Köppen-Geiger climate systems were reconstructed using the methods of Pound et al. [4]; the qualitative analysis was chosen because the present-day distributions of fungi are not as well documented as plants.

### Plant-based paleoclimate reconstructions

The probabilistic paleoclimate reconstruction model Climate Reconstruction Software (crestr) was used to define Köppen-Geiger classifications from the fossil plant assemblages of all sites (Fig. 1) [36,37]. Crestr was run in R Studio 2023.03.0+386 (Posit team 2023) following the recommended parameterization of Chevalier [37], and the resulting optima were used to define Köppen-Geiger classifications following Belda et al. [38] and Beck et al. [36]. Full results with 0.5 and 0.95 uncertainties, comparison with previously published paleoclimate reconstructions, supporting references, and links to the crestr script are available in the Supplementary Materials (SI5 and SI6).

## Data Availability

All data are available within the Supplementary Materials.

## Supplementary Materials

Supplementary 1 SI1 to SI6 References [39–107]
