# Supplementary material for: Summer-Wet Hydrologic Cycle during the Middle Miocene of the United States: New Evidence from Fossil Fungi
Source: Research (Wash D C). 2024 Sep 24;7:0481. doi: 10.34133/research.0481 (PMC11420851; doi:10.34133/research.0481)

Supplementary Information 1 – Stratigraphic Columns for the Clarkia Konservat-Lagerstätte, Alum Bluff, and Bouie River sites

SI1.1 Clarkia Konservat-Lagerstätte

Clarkia Konservat-Lagerstätte is exposed at locality P-33 (Racetrack Site) near Clarkia, Idaho and P-37 (Rember Homestead) near Fernwood, Idaho. Multiple paleontological, paleobotanical, and chronostratigraphic studies have been completed in this area[8-10,14,15,39-46], which together both constrain the age of the deposit and define the preservation history of the site. In addition to its well-known leaf flora, many of which preserve original colors and fragmentary genetic material [47,48], the palynomorph recovery is incredibly rich, with over a million grains per gram recovery in even slim samples. Sedimentologically, the site is a laminated silty claystone with evidence of turbidite-style deposition in deeper portions of the lake; these sediments have been assigned to the Latah Formation. Organic matter preservation is best in the lower, more clay-rich and waterlogged portions of both sites and is especially poor in the upper portion of P-37, where a modern fungus is using the fossil leaves as a food source (see figure 3 of Head et al.[49]).
Accumulation of sediments at Paleolake Clarkia began when the ancestral St. Maries River was dammed by a basalt flow, 15.895±0.019 MA [43,45,46]and continued until an indeterminate following emplacement of ash RA-4, 15.31±0.17 MA [42]. A best estimate for this time based on Höfig et al.'s[40] age-depth model for P-33 of 5-13 mm/annual cycle over 7.5 meters, this may be an additional 724-278.5 years. Thus, sediments at Clarkia represents less than a million years of deposition, roughly coincident with the middle of the Monterrey excursion[1].
Exposed portions of the Clarkia Konservat-Lagerstätte at the time of study comprise 22.8-m of sediment: 12.7-m at P-33, and 10.1-m at P-37 (Fig. SI1.1). An approximately 8-m gap exists between the two sections. While the exposures were sampled at 10-cm intervals, this study focusses on 30 samples, on 1-m spacing through the exposure lower 2/3 of the exposure, however, near the top of the exposure in a region of P-37 known as 'Stefanie's Pit', sample spacing is finer in an attempt to replicate results from the Pipis[8] thesis and examine the impact of emplacement of ash RA-3 on fungal preservation; no impact was noted. Samples were taken from freshly excavated vertical trenches and collected primarily by pounding 15-cm long, 2.5-cm diameter PVC pipes to a depth of 10 cm in the exposure; a grain scoop with a similar depth was used where tubes were impractical or unavailable.

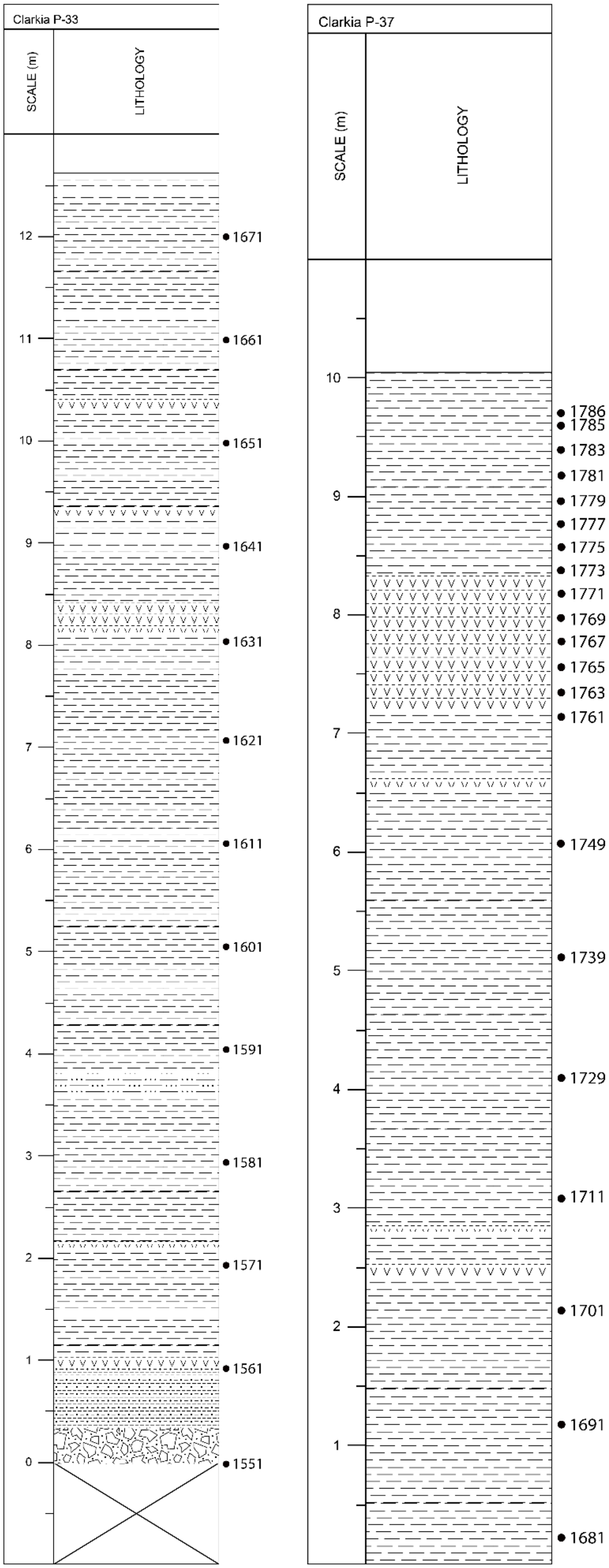

Fig. S1.1

Measured stratigraphic columns showing sampling points for sites P-33 (left) and P-37 (right) within the Latah Formation.

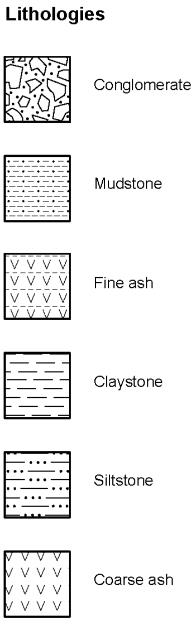

SI1.2 Alum Bluff

The Alum Bluff site is a world-famous invertebrate, vertebrate, and plant paleontological locality within the Apalachicola Bluffs and Ravines Nature Preserve in Liberty County, Florida. The bluff, located on the eastern, cutbank side of the Apalachicola River, exposes a series of truncated marine, marginal marine, and terrestrial units that record three distinct pulses of deposition, assigned to the Chipola Formation, the Fort Preston Formation, and the Jackson Formation within the Alum Bluff Group[50,51]. The Fort Preston Fm., target of this study, is separated from the underlying Chipola Fm. by a scour surface and a basal conglomerate. It is a composed of approximately 6-m of yellow and white, planar- to cross-bedded, silt- and clay-rich, weakly carbonate cemented sand with thin discontinuous grey-brown clay lenses that occur both along cross-beds and planar bedding planes. The entire unit is fossiliferous, with both shell fragments and plant debris occurring on cross-bed foresets, and fine rootlets penetrating bedding along many horizons. Fossil plants and wood are best known from the upper portion of the section, with five distinct plant horizons within a half-meter of each other near the top of the unit [52], which may be locally cut out by the disconformable contact with the overlying Jackson Formation. Most recent published chronostratigraphy using a combination of mammalian biostratigraphy and $^{87}Sr/^{86}Sr$ of shells suggests deposition occurred after 18.3 MA and ended no earlier than 15 MA[51,53], likely with a younger base, as time is clearly missing between the Chipola and the Fort Preston Formation. This basal date may be as young as 16.2 MA[54] or 16 MA[3]. Sediments at Alum Bluff, are, therefore, roughly coeval with sediments from Clarkia.

A series of vertical trenches marching up the exposure face were excavated through the weathering rind. Samples were taken by pounding 15-cm long, 2.5-cm diameter PVC pipes to a depth of 10 cm in the exposure where possible, and as a series of parallel 3-5 cm depths where it was impossible. A series of 20 samples were spaced roughly 40 cm apart vertically through the exposure, with finer spacing targeting clay-drapes and organic-rich horizons (Figure SI1-2). In addition to newly obtained samples, slides from the Jarzen et al.[7]study were re-examined. These were collected from the edges of plant fossils obtained from clay lenses in the uppermost part of the unit, likely stratigraphically equivalent to the last sample collected in the field or just above it.

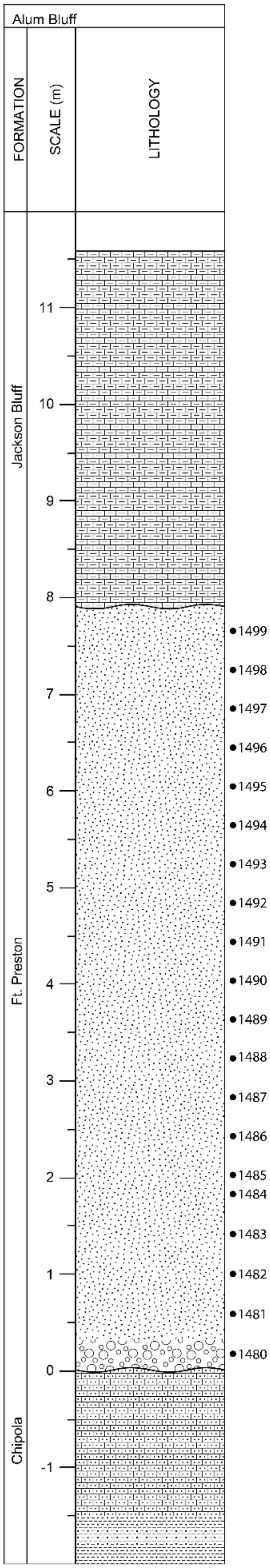

Fig. S1.2

Measured stratigraphic column for the Alum Bluff site showing sampling locations within the Ft. Preston Formation.

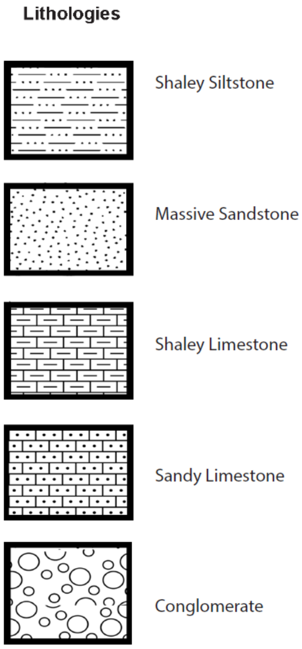

SI1.3 Bouie River Site

The Bouie River site is a relatively new paleobotanical locality (MS.18.001) situated on the southeastern bank of the Bouie River north of downtown Hattiesburg, MS[11]It is likely age-equivalent to a deposit reported by Berry[55] from Hattiesburg in his treatment of Alum Bluff. Plant macrofossils from the site were described in an initial report in 2019[11], and a single palynological sample has been processed and studied[12]. No systematic sampling of the exposure had been completed prior to the present study. The exposure consists of 2.6 meters of clay-rich siltstone and silt-rich claystone exposed along a steeply sloping riverbank. Most of the section is grey and contains abundant plant matter, ranging from wood to whole leaves in the lower section to scarce plant fragments in the upper section. Much of the site is moderately to heavily bioturbated, and bedding is largely obscured. These sediments are mapped as upper Hattiesburg Formation[11], and presently thought to range in age from 13.8-11.6 MA – they were likely deposited during the brief period of increased atmospheric pCO_2 that followed the MMCT[1]. Samples at the Bouie River site were collected as column samples taken from shallow step-trenches excavated into the bank. Roughly 50 cm of material was removed from each step to expose a fresh surface on the "riser" for sampling. This was necessary to avoid sampling in the weathering rind and to avoid any material emplaced in the soft sediments during periods of high water. The basal datum for this column was a large, lignified log embedded at river-level during sampling. The continuous column was re-described and sampled upon return to the laboratory. Samples for this study were obtained every 10 centimeters through the column.

Fig. S1.3

Measured stratigraphic column for the Bouie River site showing sampling locations within the upper Hattiesburg Formation.

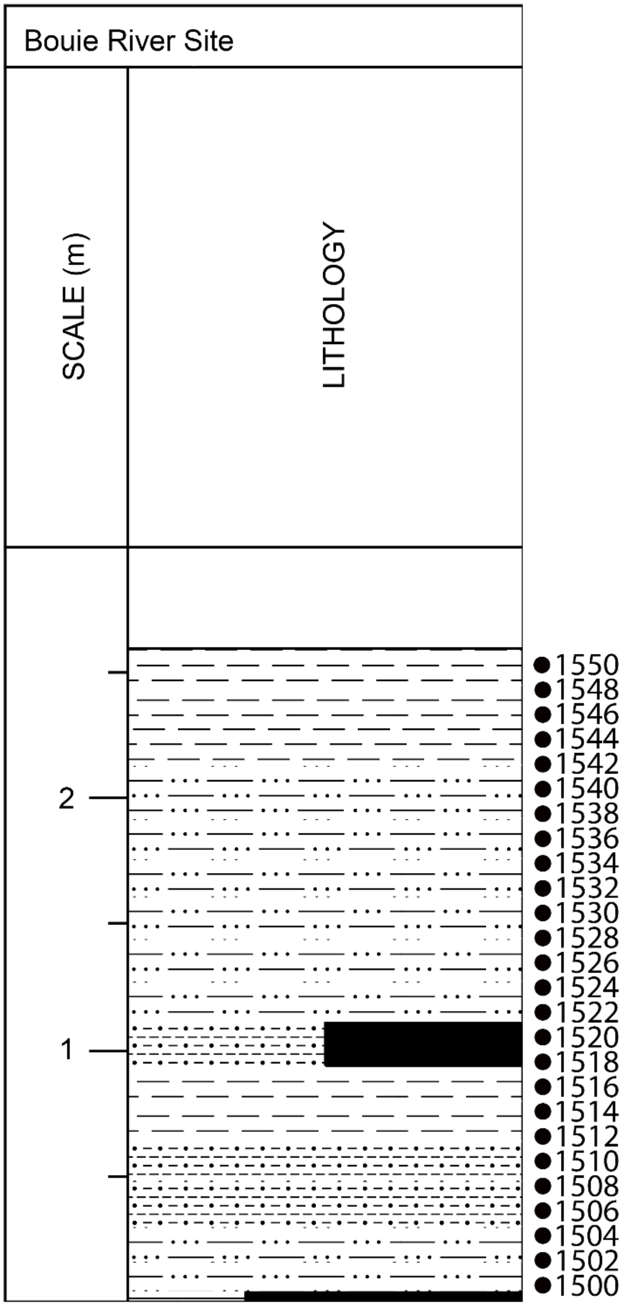

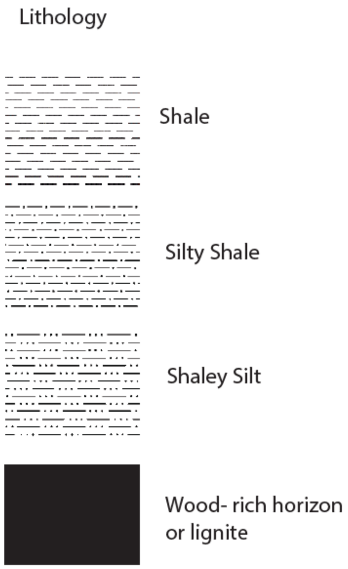

Supplement: Supplementary 1 — SI1 to SI6 References [39–107] [file research.0481.f1.zip › SI1 - Stratigraphy.pdf]
